# The Microbiome and Blood Pressure: Can Microbes Regulate Our Blood Pressure?

**DOI:** 10.3389/fped.2017.00138

**Published:** 2017-06-19

**Authors:** Souhaila Al Khodor, Bernd Reichert, Ibrahim F. Shatat

**Affiliations:** ^1^Immunology, Inflammation and Metabolism, Division of Translational Medicine, SIDRA Medical and Research Center, Doha, Qatar; ^2^Division of Neonatology, SIDRA Medical and Research Center, Doha, Qatar; ^3^Weill Cornell Medical College, New York, NY, United States; ^4^Pediatric Nephrology and Hypertension, SIDRA Medical and Research Center, Doha, Qatar; ^5^Medical University of South Carolina, Charleston, SC, United States

**Keywords:** hypertension, dysbiosis, microbiota, lifestyle, blood pressure, short-chain fatty acid, microbial metabolites

## Abstract

The surfaces of the human body are heavily populated by a highly diverse microbial ecosystem termed the microbiota. The largest and richest among these highly heterogeneous populations of microbes is the gut microbiota. The collection of microbes and their genes, called the microbiome, has been studied intensely through the past few years using novel metagenomics, metatranscriptomics, and metabolomics approaches. This has enhanced our understanding of how the microbiome affects our metabolic, immunologic, neurologic, and endocrine homeostasis. Hypertension is a leading cause of cardiovascular disease worldwide; it contributes to stroke, heart disease, kidney failure, premature death, and disability. Recently, studies in humans and animals have shown that alterations in microbiota and its metabolites are associated with hypertension and atherosclerosis. In this review, we compile the recent findings and hypotheses describing the interplay between the microbiome and blood pressure, and we highlight some prospects by which utilization of microbiome-related techniques may be incorporated to better understand the pathophysiology and treatment of hypertension.

## Background

The human microbiota is a mixed community of microorganisms composed of bacteria, viruses, archaea, and eukaryotic microbes that coinhabit the human body surfaces (1). The collection of those microbes and their genes is named the human microbiome (2).

Multiple studies have shown that each body site is characterized by unique ecological communities of microbial species (1) and each person has a unique microbiome (3). Those interpersonal variabilities are likely related to differences in our genetic background, origin, geographical location, age, life style, diet, and early exposure to various microbes, as well as exposure to antibiotics or probiotics (3). The microbiome composition is also affected by early life events; including delivery mode, gestational age, hospitalization, and the method of feeding (4).

Hypertension is a global public health problem and contributes to the burden of heart disease, stroke, kidney failure, premature death, and disability. It is considered the most prevalent modifiable cardiovascular disease (CVD) risk factor. High blood pressure affects 1.13 billion people worldwide. In the US, 75 million American adults (29%) suffer from high blood pressure, which is around one out of three adults (5). It is also estimated that more than 3% of children suffer from hypertension; this number increases in obese children, since the prevalence of primary hypertension rises progressively with increases in BMI percentile from less than the fifth percentile (2%) to more than the 95th percentile (11%) (6). Globally, 42 million preschool children were overweight in 2013. The prevalence of obesity in the US is about 17%; it affects about 12.7 million children and adolescents (7, 8). It is important to mention that while obesity is associated with increased prevalence of hypertension, not all obese children are hypertensive.

Recently, multiple animal and human studies have examined the relationship between the gut and the oral microbiome and blood pressure (9–11). These studies aimed to explore different hypotheses linking the microbiota and its metabolites to blood pressure. Here, we provide an overview of the literature and discuss the proposed mechanisms. We also discuss potential microbiota-altering therapies and lifestyle modifications and their effect on blood pressure.

## The Microbiome in Health and Disease

Our microbiota is highly dynamic and continuously changing. This is in part a reflection of age-related changes like growth and development, where the highest variation takes place during childhood but later decreases with age (12). Newborns’ and infants’ microbiota differentiates and becomes distinct and site-specific as they grow older (13). The microbiota in infants less than 6 months old is different from that of older infants, where by the age of 3 years, a child’s microbiome is highly similar to that of an adult and is considered relatively stable (14, 15). However, the microbiome composition remains subject to changes related to any disease, change of diet, use of antibiotics, and in response to major life events like pregnancy and puberty (16–18).

Microbiota composition is also determined by the physical characteristics and chemical properties of the site that is being colonized (19). Therefore, the primary determinant of community composition is the anatomical location: within the same habitat, interpersonal variation is significant (20) and is more complex than the temporal variability observed in multiple sites within the same individual (21). Those site-specific signatures of the microbiome help elucidate the many changes associated with health and disease.

### Tools Used to Study the Microbiome

**Figure 1:**
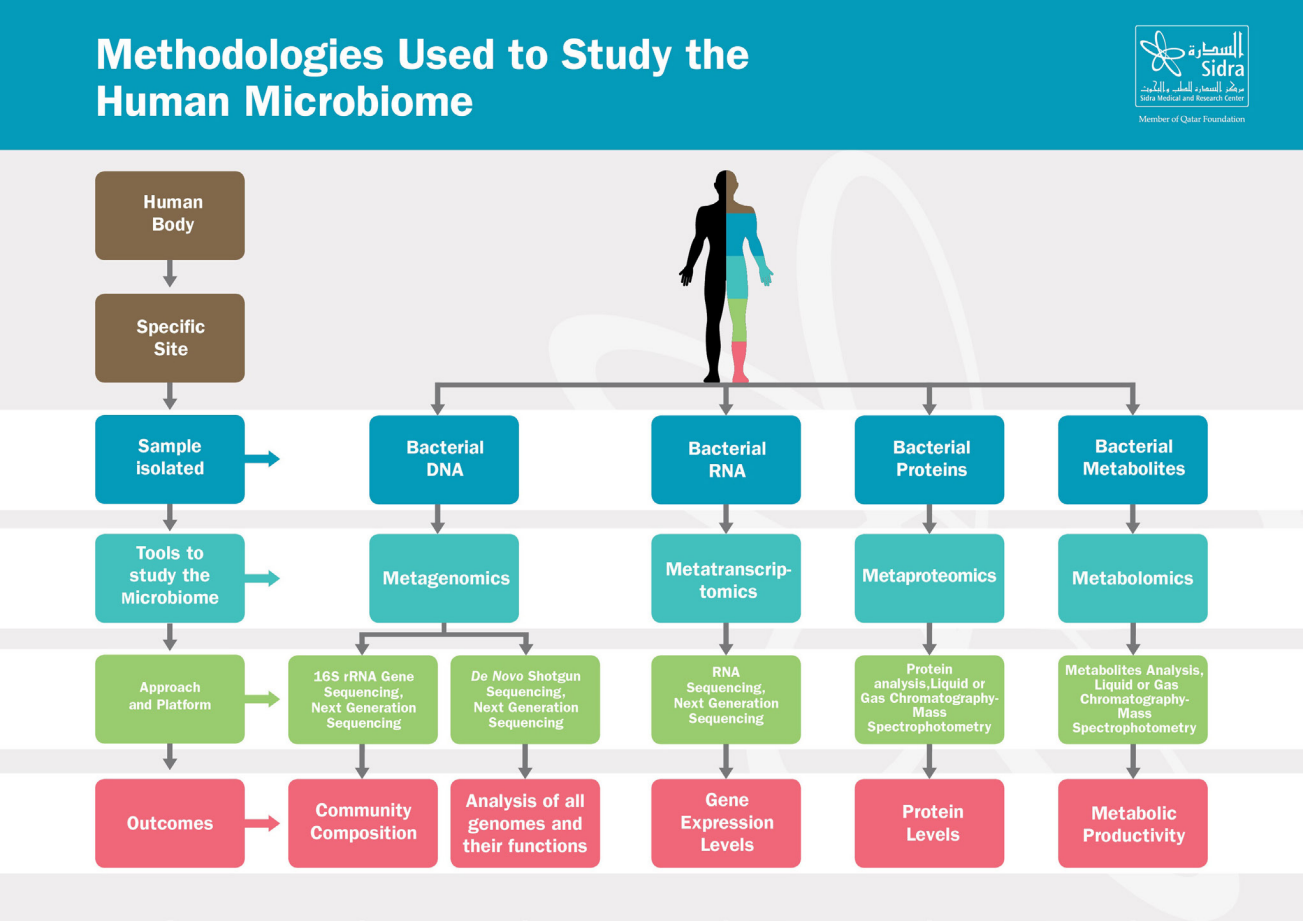
Methodologies used to study the human microbiome. The human body harbors large amounts of microbes that vary from one site to another. From each specific site, microbes can be studied in various ways depending on the samples isolated. Those tools range from metagenomics, metatranscriptomics to metaproteomics and metabolomics. Based on the approach and platform used, results include composition of the microbial communities, microbial genes function, gene expression or proteins levels, and metabolites activities.

Recent breakthroughs in high-throughput Next Generation Sequencing techniques, summarized in Figure 1, have leveraged our understanding of the composition and the function of the microbiome and have substantially advanced our knowledge on the crucial role of the microbiome in maintaining host physiology and homeostasis (22). Those techniques range from sequencing of the 16S rRNA-encoding genes, used to characterize the microbial phylogenetic composition of a sample collected from a specific body site, to the shotgun metagenomic approaches, used to identify all the genomes of microbes coexisting in the same site along with their biological functions (23–25). In addition to genomic sequencing-based analysis, other methods have been developed to study the microbial transcriptome, proteome, and metabolome, as they provide additional information at successive levels of microbial physiology (26). Metabolomics aim to study the metabolic functions by which the microbiota contributes to the human physiology; those functions include energy harvest, bile acid transformations, choline transformation, and the production of short-chain fatty acids (SCFAs), vitamins, and amino acids (27).

Those tools have tremendously contributed to our current knowledge about the microbiota and their metabolites (22, 27). In recent years, the microbiome was shown to constitute a unique “fingerprint marking” that may play a role in interindividual phenotypic variation in disease presentation, prognosis, progression, and even response to treatment (15, 20).

### The Gut Microbiome

Colonization of the human gut with a wide variety of microbes takes place just before birth as evident from the diverse microbial composition of the meconium (28). Maternal microbiota contributes to the formation of the first microbial inoculum, and then soon after birth, the microbial diversity increases and converges toward an adult-like microbiota by the end of the first 3–5 years of life (29). The ecosystem of an adult human gut contains a complex array of microbes with more than 100 trillion microbial cells and more than 1,000 bacterial species (30). The composition of the gut microbiota is highly variable between subjects, as each subject harbors a unique set of microbial species, which is in general highly stable over time in healthy individuals (31). A healthy human adult gut is dominated by the Gram-positive *Firmicutes* and the Gram-negative *Bacteroidetes* (31). Most nutrients consumed through a diet are processed by an array of various human enzymes before being absorbed by the small intestine; however, the gut microbiota contributes to the metabolism of dietary fibers that are not usually digested by those enzymes (32). In the large intestine, a group of microbes including clostridial clusters IV, XIVa, *Lactobacillus*, and *Actinobacteria* (*Bifidobacterium* spp.) contribute to the fermentation of dietary plant polysaccharides or fibers, indigestible oligosaccharides, non-digested proteins, and intestinal mucin in order to produce SCFAs (acetate, propionate, and butyrate) (33). In addition to its role in food digestion, the gut microbiota plays a vital role in inhibiting pathogen-invasion by creating colonization resistance, it also contributes to the education and stimulation of the immune system, maintenance of the intestinal epithelial homeostasis and integrity, synthesis of vitamin B and vitamin K, as well as the enhancement of the motility and function of the gastrointestinal tract (GIT) (31).

### The Oral Microbiome

The oral cavity is considered one of the most highly dynamic ecosystems in the human body (34–36). 16S rRNA gene sequencing estimated 50–100 billion bacteria in the mouth, comprised of nearly 700 identified bacterial species (36–38). Up to 80% of oral bacteria are dominated by about 200 species of *Firmicutes* and *Proteobacteria*, along with *Bacteroidetes, Actinobacteria*, and *Fusobacteria* totaling upwards of 95% of all identified oral microbiota (36–38). Further diversity exists between the varied niches of the mouth (34), such as the tooth surface, gingiva, hard and soft palate, and even regionally on the tongue (34). Within this complexity, an ever-growing number of specific microbial taxa have been associated with both oral and systemic diseases (39). For instance, saccharolytic (sugar metabolizing) bacteria, such as *Streptococcus* and *Lactobacillus*, have been associated with dental caries, while proteolytic (protein metabolizing) bacteria, such as *Prevotella* and *Porphyromonas*, have been associated with periodontitis and halitosis (40). Presence of *Porphyromonas gingivalis* is shown to be associated with atherosclerosis (41), smoking (42), and several cancers (43).

### Microbial Dysbiosis and Disease

Dysbiosis is defined as the change in the composition and structure of the human microbiota of a given site, a change that may help explain why some individuals are more likely to develop certain diseases or develop a more severe form of the illness (44).

Although the relationship between microbial composition and stability to disease predisposition is not a cause-and-effect relationship, the microbiome is a contributor in many disease states, a link that has been previously overlooked. In fact, changes in the microbiome are increasingly linked to the development of several non-communicable diseases, including diabetes (45, 46), obesity (47), CVD (48, 49), cancer (50, 51), inflammatory bowel disease (IBD) (52, 53), asthma (54), and others. Recently, researchers have examined the relationship between kidney disease and the human microbiome, summarized in Ref. (55). Studies have shown a bidirectional relationship between chronic kidney disease and the gut microbiome; where microbiota-derived metabolites contribute to the progression of CKD and the state of chronic kidney disease and inflammation contributes to changes in the diversity and richness of the microbiota. Other studies showed differences in IgA disease progression and the gut microbiota composition, SCFA (derived from the microbiota) to modulate renal dysfunction in states of acute kidney injury *via* their anti-inflammatory properties, and kidney transplantation and immunosuppressive medications to significantly impact the gut microbiota composition.

It is, therefore, essential to understand the interface between the microbes and the host in any disease condition, as this may help uncover possible disease etiologies and pathogenesis. It may also be possible to use any novel microbial factors or host-related inflammatory markers as diagnostic and therapeutic targets for prediction, prevention, and treatment of some common diseases. Deciphering the possible interindividual variations in the microbial composition in various body sites and identifying the changes in site-specific microbiota composition during onset or progression of various diseases will help to pave the way toward developing microbiota-driven personalized medicine.

While large-scale studies addressed the role of the microbiome in some conditions including cancer, IBD, obesity, and diabetes, only fewer studies describing the role of the microbiome in regulating blood pressure have been published (10, 11, 56). Those studies suggested that hypertension is directly and/or indirectly linked to microbial dysbiosis, and that some microbial metabolites contribute to the regulation of blood pressure (10, 11, 56). Therefore, understanding the nature of hypertension-related microbial aberrations in various body sites may enable us to better understand the pathophysiology of high blood pressure and possibly develop personalized microbiome-based diagnostics for individuals at risk.

In this review, we discuss potential mechanisms by which the microbiota contributes to our blood pressure regulation and describe the link between dysbiosis and hypertension.

## Hypertension and the Microbiome

Blood pressure regulation is complex. Multiple physiological systems interact, influenced by the environment and genes, to maintain blood pressure (57). These include but are not limited to: the renin–angiotensin–aldosterone system, the sympathetic nervous system (SNS), the nitrate–nitrite–nitric oxide signaling pathway (NO), uric acid, endothelin, the vasopressin system, natriuretic peptides, vasodilator peptides, the tissue kallikrein–kinin system, the immune system, the adipose tissue, and adipokines (58).

Recently, multiple animal and human studies have examined the relationship between the oral and gut microbiome and blood pressure; they demonstrated a significant decrease in microbial richness and diversity in the presence of hypertension. In addition, studies have demonstrated an altered microbial composition and modified metabolite profiles, suggesting a role for microbial dysbiosis and microbial metabolites in hypertension (11). In a rat model of hypertension, the number of cecal “good bacteria” from the phylum *Bacteroidetes* is reduced, which is accompanied by a proportional increase in the number of “bad bacteria” from the phylum *Firmicutes* (11). Studies have also shown that transplant of cecal microbial content from donor hypertensive animals can reproduce hypertension in previously normotensive recipient animals (56). A third set of studies demonstrated a beneficial effect for microbial mass reduction using antibiotics on blood pressure (11). Furthermore, absence of gut microbiota was found to protect mice from angiotensin II (AngII)-induced arterial hypertension, vascular dysfunction, and hypertension-induced end-organ damage (59).

**Figure 2:**
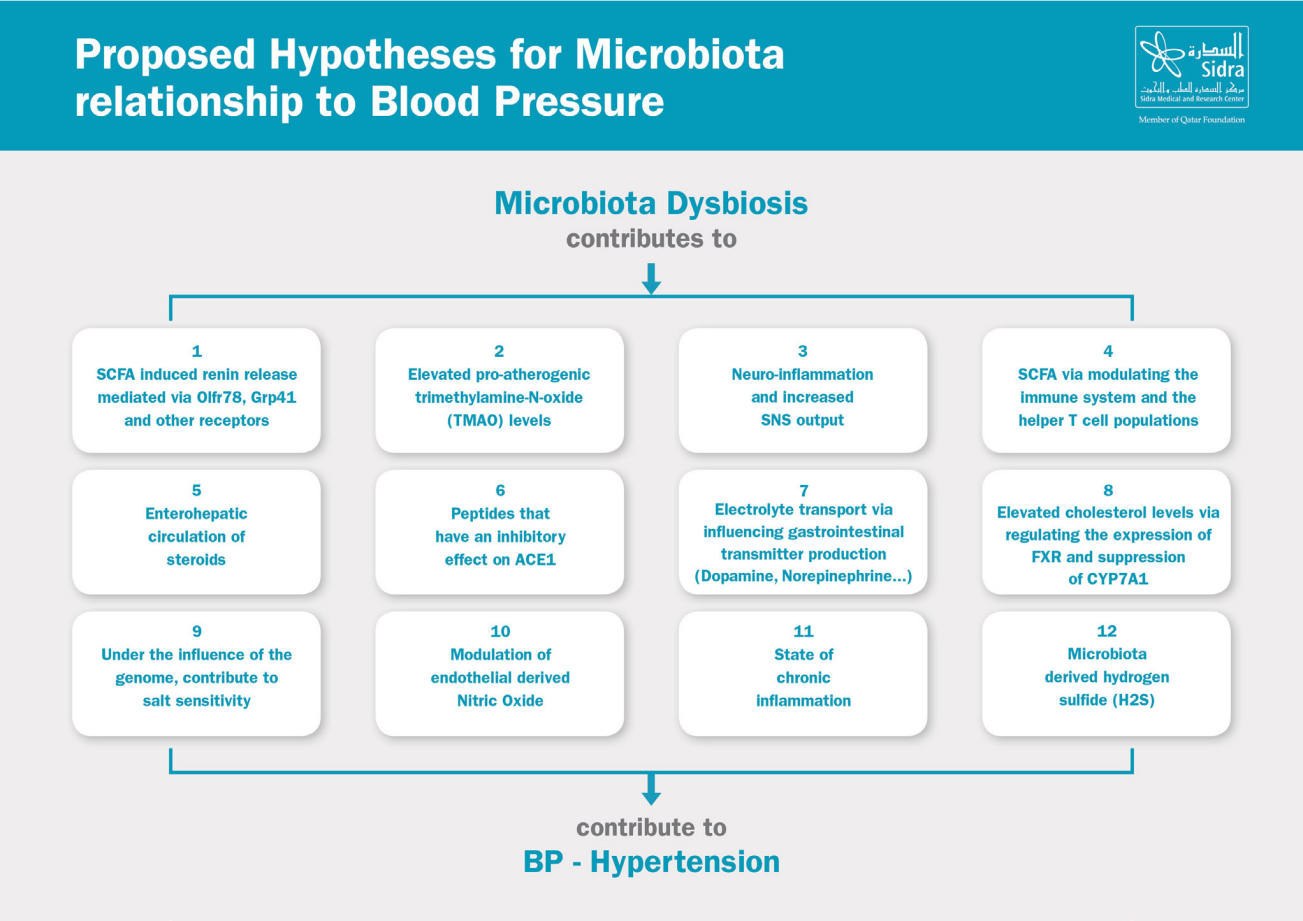
Proposed hypotheses for microbiota relationship to blood pressure. SCFA, short-chain fatty acids; SNS, sympathetic nervous system; ACE, angiotensin-converting enzyme; FXR, farnesoid X receptor.

The relationship between the microbiota and blood pressure is a complex one. Researchers have identified multiple possible hypotheses to link dysbiosis and hypertension. Many of which are indirect links that contribute to the metabolic syndrome and the overall increased cardiovascular risk. It is also important to point out that most of the studies were conducted in animal models, and many examined newly proposed hypotheses which yielded results that have yet to be reproduced. Some hypotheses focused on the association between microbial species in the microbiota and their relationship to blood pressure, whereas other hypotheses examined the role of dysbiosis in the pathogenesis (e.g., increased SNS activity), sustenance (e.g., inflammation), and worsening/progression of hypertension (e.g., endothelial dysfunction and vascular remodeling). Here, we provide an overview of the proposed hypotheses linking the microbiota to blood pressure, Figure 2.

### Microbial SCFA Metabolites Regulate Blood Pressure *via* Olfactory Receptors

Researchers have described a less rich and diverse microbiota in hypertensive compared to control subjects (11). SCFAs are products resulting from fermentation of various nutrients by the gut microbiota and are later absorbed into the blood stream (60). Members of the olfactory signaling pathway are expressed in the human kidneys. Olfr78 is an olfactory receptor that mediates renin secretion after stimulation by SCFAs (61). Other SCFA receptors including Gpr41 and Gpr43 (also called free fatty acid receptor 3 or FFAR3 and FFAR2, respectively) are also expressed in the renal vasculature. Propionate administration to Gpr41-deficient mice induced blood pressure elevation, suggesting that Gpr41 is needed to counterbalance the pressor response to SCFA (10).

### Increased Risk of Atherosclerosis from Microbiota-Generated Trimethylamine-*N*-Oxide (TMAO)

Another indirect connection between the gut microbiome composition and hypertension derives from the role of the gut microbes in the metabolism of choline and phosphatidylcholine to trimethylamine (TMA), which is further metabolized to the pro-atherogenic species, TMAO (62). Koeth and colleagues showed that the metabolism of dietary l-carnitine, a TMA abundant in red meat, by the intestinal microbiota results in the production of TMAO and accelerates the development and progression of atherosclerosis in mice (62). Recently, a study in a healthy human volunteer showed that microbiota in the small intestine generated the phosphatidylcholine breakdown product TMA (63). The resulting TMAO was suppressed by topical-acting antibiotics (63). It is important to point out that this link is with atherosclerosis and not directly to hypertension.

### Microbiota-Induced Neuroinflammation and Increased Sympathetic Activity

Vagal nerve stimulation and blocking sympathetic derive prevent breakdown of the intestinal lumen–blood barrier and enhance epithelia cell barrier function (64, 65). Santisteban and colleagues hypothesized that hypertensive stimuli (such as Ang II, salt, and stress) trigger autonomic neural pathways resulting in increases in sympathetic and dampening of the parasympathetic activities which in turn contribute to the overall increase in blood pressure (66). Increased sympathetic nervous activity to the gut could result in increased gut permeability, gut inflammation, and dysbiosis, leading to an imbalance in the microbial-derived metabolites in the plasma, possibly contributing to chronic inflammation and sustained hypertension.

### Modulation of Blood Pressure *via* the Effects of the Microbiota on the Immune System

Researchers have proposed a brain–gut–bone marrow axis in which sympathetic activity to the bone marrow induces mobilization of hematopoietic stem cells (66). In this hypothesis, hematopoietic stem cells may migrate to the brain or to the gut and contribute to local inflammation and immune responses. This may further increase the sympathetic activity and contribute to blood pressure elevation. On the other hand, SCFAs, such as acetate and butyrate, have been shown to have anti-inflammatory effects on myeloid and intestinal epithelial cells (67). Recently, Kim and colleagues reported marked decreases in microbial richness and diversity in hypertensive patients and also observed marked differences in circulating inflammatory cells in hypertensive individuals compared to controls (68); T-helper 17 cells were particularly relevant because activation of these cells is regulated by gut-intrinsic mechanisms, and their increase may be a result of dysbiosis in hypertension (68). In germ-free mice, the absence of gut microbiota seems to protect the animals from AngII-induced arterial hypertension, vascular dysfunction, and hypertension-induced end-organ damage. This protection appears to be mediated by inhibiting the accumulation of the inflammatory myelomonocytic cells in the vasculature and altered cytokine signaling (59).

### Role of the Microbiota in the Enterohepatic Circulation of Steroids

Another intriguing hypothesis involves the role of the gut microbiota in the enterohepatic circulation of steroids. Using antibiotic therapy, the bacterial flora of rats was modified to interrupt the enterohepatic circulation of steroids excreted in bile. Antibiotic and corticosterone were administered simultaneously to these animals. After 5 days, rats receiving both steroids and antibiotics had an average elevation of 9.2 mmHg in their blood pressure compared with 24.6 mmHg in rats given steroid alone. These findings are consistent with the possibility that metabolites of steroids, when reabsorbed in the enterohepatic circulation, contribute to the physiological response to exogenous steroids (69). Further studies are needed to examine the role of gut-derived steroids.

### Inhibition of Angiotensin 1-Converting Enzyme

Nakamura and colleagues (70) reported a significant decrease in the systolic blood pressure in rats fed sour milk (contains the two tripeptides Val–Pro–Pro and Ile–Pro–Pro). Research on the health effects of pasteurized sour milk, which is fermented by a starter culture containing *Lactobacillus helveticus* as the predominant microorganism, indicated that antihypertensive effects and angiotensin-I converting enzyme inhibitory peptides are present in sour milk (71, 72). On the other hand, researchers have shown ACE2 has a RAS-independent function, regulating intestinal amino acid homeostasis, expression of antimicrobial peptides, and the ecology of the gut microbiome (73).

### Electrolyte Transport *via* Influencing Gastrointestinal Transmitter Production

The gut microbiota can influence the ability of the gastric and intestinal enterochromaffin cells to produce serotonin, dopamine, and norepinephrine. These transmitters have been found to influence Na–K ATPase and electrolyte transport in the intestine (74). It is still unclear whether enterochromaffin cell-derived transmitter effects are clinically significant in blood pressure regulation. Further studies are needed to examine the role of gastrointestinal transmitter production on blood pressure regulation.

### Elevated Cholesterol Levels *via* Regulating the Expression of RXR and Suppression of CYP7A1

Cholesterol is the precursor to bile acids synthesis in the liver. Bile acids are further metabolized by the gut microbiota into secondary bile acids. In the ileum and liver, nuclear farnesoid X receptor (FXR) plays a key role in the bile acid synthesis; when activated, it exerts negative feedback to control bile acid synthesis (75). Gut microbiota may indirectly play a role in the increased risk of atherosclerotic disease by increasing cholesterol levels; this may be attributed to its role in reducing the levels of tauro-beta-muricholic acid, a FXR antagonist, as well as by suppressing the rate-limiting enzyme CYP7A1 in bile acid synthesis from cholesterol (75). It is important to point out that this link is with atherosclerosis and increased cholesterol levels and not directly to hypertension.

### Under the Influence of the Host Genome, Microbiota May Contribute to Salt Sensitivity

Mell and colleagues hypothesized that the interaction between the host and the gut microbiota influences the development of salt-sensitive hypertension (76). They reported differences in the gut (cecal) microbiota composition between the salt-sensitive (S) and the salt-resistant (R) Dahl rats. After a single bolus of R rat cecal content to S rats, they showed exacerbated hypertension in high salt-fed S rats, with systolic blood pressure to be consistently and significantly elevated during the rest of the recipient rat life, which also had a shorter lifespan. They speculated that this effect may be mediated *via* SCFAs, as both acetate and hetanoate were higher in the R to S transfer group (76).

### Modulation of Endothelial-Derived Nitric Oxide (NO)

It is also worth mentioning that the nitrate–nitrite–nitric oxide signaling pathway involved in the pathogenesis of hypertension is highly affected by microbial diversity through the formation of nitrite, NO, and other bioactive nitrogen oxides (77). NO is an endogenously produced, lipophilic, and diffusible molecule that exerts a diverse array of critical autocrine and paracrine signaling activities. NO acts directly on smooth muscle cells to promote relaxation and inhibits both platelet function and vascular smooth muscle cell proliferation and migration (78). Formation of nitrite and propagation of its downstream NO-signaling effects depend on the oral bacterial reduction of inorganic nitrate by a set of bacterial nitrate reductase enzymes that are largely absent from the human genome (77, 79, 80). Studies in Sprague-Dawley rats (77) and normal human volunteers (81) showed that depletion of oral bacterial nitrate reductases by chlorhexidine mouthwash correlated with a 90% decrease in oral nitrite levels in humans, along with a 25% decrease in plasma levels (*p* = 0.001), and 2–3.5 mmHg increase in blood pressure (81). Several studies in hypertensive, overweight, and other patient populations have been performed, all revealing predictable acute or chronic blood pressure reduction with varying types of nitrate supplementation (82), this beneficial effect is abolished both acutely and chronically by antimicrobial mouthwash use [reviewed in Ref. (83, 84)].

### State of Chronic Inflammation

Dysbiosis, gut wall inflammation, and increased gut wall permeability have been shown to contribute to the state of chronic systemic inflammation. Endotoxemia has been linked to the development of low-grade systemic inflammation and vascular inflammation *via* toll-like receptor-dependent mechanisms (85). In obese individuals, intestinal microbiota composition was associated with local and systemic inflammation (elevated C-reactive protein) (86).

### Microbiota-Derived Hydrogen Sulfide (H_2_S)

Microbiota, like many mammalian cells and tissues, also produce H_2_S (87). Microbes exploit this gaseous molecule as an antioxidant defense mechanism, for energy production, and for cell cycle regulation. It is estimated that 50% of fecal H_2_S is derived from bacteria, thus the total plasma H_2_S pool varies depending on the individual’s microbiota milieu in the gut. H_2_S plays a crucial role in a variety of physiological functions, including smooth muscle relaxation, oxidant regulation, inflammation, and angiogenesis (88). Hydrogen sulfide is synthesized primarily from the amino acids cysteine and homocysteine. H_2_S biosynthesis deregulation, particularly in the renal vasculature, may play a role in hypertension or possibly contribute to existing high blood pressure (89). Although theoretically plausible, it is unknown to what extent does microbiota-derived H_2_S contribute to blood pressure regulation in humans? This area is still in need of further research.

## Hypertension Effects on the Microbiota Composition and Function

While the focus of most studies was to examine how the microbiota at different body sites can modulate blood pressure, a few studies looked at the other direction of this relationship, that is, can hypertension affect our microbiota composition and cause dysbiosis? Is dysbiosis a target organ injury due to hypertension? And does dysbiosis precede, accompany or result from hypertension? It is important to point out that the majority of published studies were cross-sectional and were designed to examine associations and not to determine cause-and-effect relationships. Hypertensive animals and humans were found to have decreased microbial richness, diversity, and composition (11). Santisteban and colleagues tested the hypothesis that increased sympathetic drive to the gut in hypertensive animals is associated with increased gut wall permeability, increased inflammatory status, and microbial dysbiosis (90). Changes in gut pathology were present and were associated with alterations in microbial communities relevant to blood pressure control. However, whether gut permeability and dysbiosis played a role in the pathogenesis of hypertension or were a consequence of hypertension is still not clear. It is very well possible that the relationship between dysbiosis and hypertension is bidirectional or an amplifying one. Further studies are needed to decipher this relationship.

## Antihypertensive Medications: Gut Microbiota-Mediated Drug Interactions

The hepatic enzyme system is the key player when it comes to drug metabolism; however, the gut bacteria also exert a variety of metabolic changes to orally ingested drugs, including reductive and hydrolytic reactions. Researchers have reported gut microbiota-mediated drug interactions between multiple medications and antibiotics (91). Those interactions were mediated by alterations in the gut microbiota. The drug amlodipine’s plasma concentration area under curve was increased by up to 133% in ampicillin-treated rats. This increase in its bioavailability was attributed to the reduction of gut microbiota that usually contributes to amlodipine metabolism (92). The authors went on to caution clinicians regarding the use of antibiotics in patients treated with amlodipine. On the other hand, antibiotic treatment alone using minocycline was able to “rebalance” the microbiota and was associated with blood pressure reduction (11). Another aspect to the interaction between antihypertensive medications and the microbiota was described by Santisteban and colleagues (90). In their study, they found increased permeability and stiffness of the gut barrier, decreased levels of tight junction proteins, increased gut fibrosis, thickening of the gut muscularis layer, decreased villi length, and goblet cell loss in spontaneously hypertensive rats and in rats with AngII-induced hypertension. Treatment of SHR with captopril reduced gut permeability and completely restored fibrosis levels and thickness of the muscularis layer and only partially restored villi length (90). Some studies are underway (clinical trial NCT02188381), and more are needed to further examine the microbiota interaction and its role in the metabolism of different antihypertensive medications, as well as the effect of concomitant antibiotic treatment.

## Restoring the Balance: Current and Potential Interventions

Lifestyle changes and dietary interventions are key modifiable factors in the management of hypertension. Recently, researchers have started to examine changes in blood pressure as they manipulate the microbiome by introducing dietary and lifestyle changes.

### Lifestyle Modifications and Their Effect on Hypertension and the Microbiome

Sufficient sleep is vital for maintaining physical and mental health. Chronic sleep deficiency is related to a wide variety of diseases, including CVD and metabolic disease (93). Epidemiologic studies have established the best amount of sleep for adults as approximately 7 h and that this range correlates best with a lower prevalence of CVD and reduced risk of hypertension (94). Lately an intricate, bidirectional relationship between sleep, circadian rhythms, and the composition of the microbiome in mice was described (95, 96). Benedict et al. showed that sleep deprivation induced changes in microbial families of bacterial gut species in humans (97). Furthermore, Durgan et al. established a link between gut dysbiosis and the development of obstructive sleep apnea-induced hypertension (56). On the other hand, Zhang et al. reported that sleep restriction over several consecutive days does not overtly influence the composition of the microbiome of either rats or humans (98). Further studies are needed to examine the triangular relationship between sleep (duration and quality), blood pressure, and the microbiome.

Sedentary lifestyle is linked to poor health, increased cardiovascular, and metabolic disease risk (99). On the other hand, exercise offers a protective effect; it has a positive effect on body composition, immunity, and cardiovascular health (94, 99). Exercise affects the gut microbiome composition (100). Athletes have a more diverse gut microbiota; Clarke and colleagues showed that a positive effect exists between physical activity, increased dietary protein, and the diversity of the gut microbiome (101). Furthermore, Allen and colleagues demonstrated that different exercise modalities (forced and voluntary) can evoke changes in richness and evenness in the microbiome at varying body sites (102). It remains unclear whether the effect of physical activity on the microbiome is independent of any accompanying adjustment of dietary intake (mainly protein) (100).

### Diet and Its Effect on Hypertension and the Microbiome

Multiple dietary components have been shown to affect blood pressure (103), and various studies have examined the effect of manipulating those components on the blood pressure (104). The Dietary Approaches to Stop Hypertension (DASH) diet is one of the interventions used to reduce blood pressure (98). This diet is rich in fruits and vegetables, as well as low-fat dairy, and at the same time has a low content of saturated and total fat (105). DASH-sodium trial demonstrated significant dose–response decreases in blood pressure when the DASH diet and sodium restriction were combined (105, 106). This reduction in blood pressure was accompanied by 30 and 20% risk reduction of CVD after a long-term follow-up for 15–20 years, respectively (107). It is possible that components (high fiber, dairy) of such interventions alter the microbiota in favor of a more balanced one and contribute to its blood pressure-lowering effects (70).

### Food Supplements and Their Effect on Hypertension and the Microbiome

#### Prebiotics

Prebiotics are non-digestible food ingredients that escape digestion in the upper part of the GIT, only to be available for breakdown and fermentation by the gut microbiota within the lower parts of the GIT (108). Most prebiotics are derived from plants. Their role in lowering the CVD risk has been attributed to their ability to lower serum lipid and cholesterol levels (109). Population studies indicate that higher dietary fiber intake was significantly associated with a lower risk of obesity and hypertension (110). Another possible mechanism by which prebiotics could regulate blood pressure is through the attenuation of insulin resistance (111). Additionally, prebiotics have also been reported to reduce the risk of hypertension by improving the absorption of minerals such as calcium in the GIT (112).

#### Probiotics

Probiotics are living microorganisms that confer a health benefit on the host when administered in sufficient amounts (113). Some probiotic strains exhibit antihypertensive effects: for example, consumption of a dairy product mixture, including *Enterococcus faecium* and two strains of *Streptococcus thermophiles*, for 8 weeks lowered systolic blood pressure (114). Administration of *Lactobacillus plantarum* 299v for 6 weeks was also found to reduce systolic blood pressure in heavy smokers (115). Furthermore, consumption of probiotics-fermented potato yogurt could reduce hypertension-induced cardiac myocyte apoptosis in hypertensive rats and, therefore, can promote cardiac protection against hypertension (116). *Lactobacillus casei* and *Streptococcus thermophilus* TMC 1543 were also proven to lower systolic blood pressure and risk factors that caused ischemic heart disease (117).

#### Synbiotics

Synbiotics are nutritional supplements containing both probiotics and prebiotics in a form of synergy (118). Synbiotics improved survival and distribution of microbial supplements within the GIT by facilitating selective stimulation and activation of growth and metabolism of probiotics (118). Just like prebiotics and probiotics, synbiotics can modulate the gut metabolic activities without modifying the overall structure. Predominant strains of probiotics used in synbiotic preparations include Lacbobacilli, Bifidobacteria species, *Saccharomyces boulardii*, and *Bacillus coagulans*, whereas Oligosaccharides, inulin, and other dietary fibers from natural sources form the basis of the prebiotic component. It is worth mentioning that an animal study examining the role of the synbiotic dietary supplement of *Lactobacillus plantarum* HEAL19 together with fermented blueberry was not effective in lowering blood pressure in hypertensive rats (119). To our knowledge, there are no human trials evaluating the effects of synbiotics on hypertension, such trials are warranted.

#### Xenobiotics

Xenobiotics are chemicals or substances that are foreign to an organism or biological system. They are not nutrients and enter the body through ingestion, inhalation, or dermal exposure (120). Xenobiotics have the potential to induce gut dysbiosis and influence disease states. Previously published reviews elegantly shed the light on potential mechanisms that link the human gut microbiome to the efficacy and toxicity of xenobiotics (drugs, dietary compounds, and environmental toxins), even after short periods of exposure (121, 122). However, more research is needed to understand the interactions between xenobiotics, blood pressure, and the gut microbiome.

#### Fecal Microbiota Transplants (FMT)

In FMT, the fecal matter is collected from a tested donor, then blended with saline or other solutions, filtered, and drained and later administered to the recipient *via* colonoscopy, endoscopy, sigmoidoscopy, or enema.

As the use of FMT in the management of severe or recurrent *Clostridium difficile* infection is becoming well established (123, 124) and its use in the treatment of IBD, especially ulcerative colitis, is being intensely studied (125), FMT is being increasingly evaluated for use in other areas. Vrieze and colleagues reported improved insulin sensitivity by transfer of microbiota from lean donors to individuals with metabolic syndrome (126). This result gives hope that FMT may become part of treatment regimens for metabolic syndrome and resistant hypertension in the future. Studies have also shown that transplant of cecal microbial content from donor hypertensive animals can reproduce hypertension in previously normotensive recipient animals (56); whether this can be reproduced in humans and whether reverse transplantation will achieve blood pressure reduction in hypertensive subjects remain to be tested.

More work is required to establish the effect of FMT in resistant hypertension. This will include careful evaluation, screening and donor selection, transplant composition, as well as mode of delivery of the transplant (127, 128). Also, it is not known whether the microbiota manipulation can be sustained without continuous application or the need for a concurrent change in dietary or lifestyle habits.

## Conclusion

Studies have described how dysbiosis may modulate blood pressure and contribute to CVD. While most studies were performed using animal models, a few studies were conducted in adult hypertensive subjects and none were conducted in children. Given the differences in the gut microbiota composition between children and adults, there is a pressing need for more studies in the pediatric population; it is necessary to characterize the microbiome profile in hypertensive and obese hypertensive children compared to their siblings and their healthy counterparts.

Understanding the nature of hypertension-related microbial aberrations in various body sites, may enable future development of personalized microbiome-based diagnostics and therapies for individuals at risk. Identifying specific microbial signatures associated with the high-risk population may potentially serve as a biomarker to develop non-invasive diagnostics tools. Multiple promising interventions have been described to restore a more balanced microbiome; such treatments need to be further examined in a systematic way to evaluate their potential in lowering blood pressure through modulation of the microbiota. It is worth mentioning that clinical trial NCT02188381 is currently recruiting participants to study gut microbiota involvement in the neuroinflammation-mediated initiation and establishment of resistant hypertension, as well as the possible beneficial role of minocycline therapy on outcomes in resistant hypertension.

Large, prospective clinical trials to establish a more definitive relationship between dysbiosis and high blood pressure and to identify specific microbial signatures in hypertensive subjects are needed before deploying any therapies targeted at altering the microbiota composition.

## Author Contributions

All authors (IS, BR, and SA) have equally contributed to the manuscript.

## Conflict of Interest Statement

The authors declare that the research was conducted in the absence of any commercial or financial relationships that could be construed as a potential conflict of interest.
